# What factors influence continuous usage intention of head-mounted display-based virtual reality content? A cross-sectional survey

**DOI:** 10.4069/kjwhn.2023.09.11.02

**Published:** 2023-09-26

**Authors:** JeongSil Choi, Heakyung Moon, Mijeong Park

**Affiliations:** 1 College of Nursing, Gachon University, Incheon, Korea; 2 Department of Nursing, Hoseo University, Asan, Korea

**Keywords:** Head-mounted display, Health services, Intention, Virtual reality

## Abstract

**Purpose:**

The aim of this study was to explore the continuous usage intention of head-mounted display-based virtual reality (HMD-based VR) content among college students. The study also sought to understand how this intention is influenced by factors related to personal cognition, social aspects, VR content, and HMD-related elements.

**Methods:**

This descriptive correlational study used a self-report questionnaire to survey 217 students from two universities in Korea who had prior experience with HMD-based VR content.

**Results:**

The mean score for continuous usage intention of HMD-based VR content was 2.59±0.57 points (range, 1–5 points). Regarding the average frequency of HMD-based VR content usage, 64.5% of participants reported using it 1 to 2 times, while 91.7% indicated a total HMD-based VR usage period of less than 6 months. Factors such as personal cognition, VR content, social aspects, and HMD-related elements had explanatory power of 35.1%, 10.7%, 4.4%, and 2.5%, respectively, for the continuous usage intention of HMD-based VR content. Additionally, engagement (β=.45, *p*<.001), influential others (β=.37, *p*<.001), environmental support (β=–.18, *p*=.030), and cyber sickness (β=–.21, *p*=.001) were identified as having a significant influence.

**Conclusion:**

When developing HMD-based VR content, strategies to improve users’ personal cognition should be included. Additionally, it is necessary to develop strategies that enhance enjoyment and interest in the content, while also facilitating ongoing social support. Furthermore, coping strategies should be devised that take into account cyber sickness, a potential side effect of these devices.

## Introduction

The integration of information and communications technology (ICT) with services and content from various industries has ushered in new changes and experiences for users. Notably, the availability of related hardware has propelled virtual reality (VR) to the forefront, offering content for gaming, educational, and training experiences across diverse industries [1]. The immersive capabilities of VR facilitate user engagement in achieving specific goals by enabling active interaction with virtual three-dimensional (3D) content [2]. In the healthcare field, VR is being innovatively utilized for purposes such as medical staff training, pain management, 3D disease visualization, remote early diagnosis, and patient education. Reports suggest that VR has had a positive impact as a novel educational and training tool, as well as a promising aid for disease diagnosis and treatment [3-5].

To maximize leverage the immersive capabilities of VR, users are required to don equipment such as a head-mounted display (HMD), data glove, and data suit. The HMD, in particular, enhances immersion by isolating the user from their external environment, thereby enabling a more authentic VR experience [2]. This makes it a popular choice when developing VR programs for the healthcare sector. However, the widespread commercial adoption of HMD-based VR has been impeded by uncomfortable side effects, such as cyber sickness, which can occur during the VR experience [2,6]. Furthermore, questions persist about the sustainability of the novelty effect, which typically boosts performance in the early stages [7]. The challenges associated with HMD-based VR usage have been a topic of discussion for some time, with no straightforward solutions on the horizon [8,9]. Additionally, the majority of studies have primarily focused on cyber sickness [2,8,9], leading to a misconception that advancements in virtual headset technology are the sole solution to the issues associated with HMD. This could potentially hinder the exploration of diverse methods that could encourage sustained use of HMD-based VR content.

Most studies have examined technology acceptance in relation to VR use, and it is difficult to find studies that have attempted to identify and incorporate the needs of consumers who use HMD-based VR. HMD-based VR is not merely a single form of technology, but also a tool that can enhance user satisfaction and promote health benefits through experiential effects. Therefore, the testing and development of HMD-based VR should not be solely based on findings regarding technology acceptance. Particularly in the healthcare sector, where the focus is on the emotional and behavioral aspects of humans, it is crucial to identify and meet the diverse needs of service consumers for the HMD-based VR technology service market to expand and flourish.

Thus, this study aimed to offer strategic insights for the creation of technology that can enhance user satisfaction and encourage ongoing use. This is achieved by considering relevant factors from the development phase of HMD-based VR content, with a focus on health promotion in the healthcare sector. For the successful completion of a specific task, it is crucial to embrace the technology or service and identify the factors that enable its sustainability. Factors influencing the use and ongoing intention to use ICT, such as mobile apps, can be broadly categorized into personal and technological characteristics [10]. Trice and Treacy [11] have noted that key factors affecting personal ICT usage include design and implementation process variables (e.g., overall implementation strategy, accuracy of user expectations, and top management support), information system characteristics (e.g., response time, accuracy, relevance, stability, and security), individual differences (e.g., age, experience, educational level, and cognitive style), and task characteristics (e.g., complexity and uncertainty). In light of this, the current study categorized the various characteristics that could influence the ongoing intention to use HMD-based VR content into four groups: personal cognition, social factors, VR content, and HMD-related factors. The aim was to identify the specific influence of each of these factors.

The specific objectives of this study were as follows. First, we aimed to identify personal characteristics associated with continuous usage intention among individuals with experience of using HMD-based VR content. Second, we sought to determine the degree of personal cognition (self-efficacy and innovative propensity), social factors (influential others and environmental support), VR content factors (engagement, functionality, aesthetics, and presence), and HMD-related factors (cyber sickness and physical discomfort) in relation to the continuous usage intention of HMD-based VR content. Third, we aimed to identify the distribution of levels of personal cognition, social factors, VR content factors, and HMD-related factors according to individuals’ experiences with HMD-based VR content. Fourth, we investigated the correlations between the continuous usage intention of HMD-based VR content and personal cognition, social factors, VR content factors, and HMD-related factors. Fifth, we aimed to determine the influence of personal cognition, social factors, VR content factors, and HMD-related factors on the continuous usage intention of HMD-based VR content.

## Methods

Ethics statement: This study was approved by the Institutional Review Board of Gachon University (1044396-201905-HR-074-01). Informed consent was obtained from the participants.

### Study design

This descriptive correlational study aimed to explore the influence of personal cognition (self-efficacy and innovative propensity), social (influential others and environmental support), VR content (engagement, functionality, aesthetics, and presence), and HMD-related factors (cyber sickness and physical discomfort) on the continuous usage intention of HMD-based VR content. This study adhered to the STROBE (Strengthening the Reporting of Observational Studies in Epidemiology; http://www.strobe-statement.org/) reporting guidelines.

### Study participants

Participants were selected through convenience sampling from two universities in cities Asan and Incheon. These students had prior experience with HMD-based VR content and were recruited in December 2019. To boost the participation rate, trained research assistants approached students in the universities’ libraries and cafeterias, where they verbally explained the purpose and methods of the research. Eligible and willing participants were then asked to complete a questionnaire in a private setting, seal it in an unmarked envelope, and submit it to the researcher. Upon completion of the questionnaire, participants received a small token of appreciation. Based on similar previous studies [12,13], the minimum sample size needed for linear multiple regression analysis was determined to be 167, using α=0.05, a medium effect size f=0.15, power=0.95, and 19 predictors. To account for the possibility of incomplete responses, a total of 240 students were recruited. Of these, 239 surveys were received (a response rate of 99.6%). After discarding questionnaires with incomplete or insincere responses, the questionnaires from 217 participants were used for the analysis.

### Measurements

Continuous usage intention, personal cognition-related factors, social factors, VR content factors, and HMD-related factors were measured (55 items in total, taking 20 minutes). The use of all the measurements in this study was approved by the original developers and translators of the Korean versions.

#### Continuous usage intention of head-mounted display-based virtual reality content

To measure the continuous usage intention of HMD-based VR content, three items from the Smartphone App Use Intention Scale developed by Park et al. [13] were partially revised in accordance with the objective of the present study (i.e., revised to “I have the intention to continue using HMD-based VR content”). Each item in the instrument is scored on a 5-point Likert scale (1, “not at all” to 5, “very much so”), with higher mean scores indicating higher continuous usage intention of HMD-based VR content (possible range, 1–5 points). The reliability of the instrument was shown by a Cronbach’s α of .73 in the study by Park et al. [13] and .91 in the present study.

#### Personal cognition factors

**Self-efficacy:** To measure confidence in successfully using HMD-based VR content, four items from the Self-Efficacy Regarding Smartphone App Scale developed by Park et al. [13] were partially revised in accordance with the objective of the present study (i.e., revised to “I can use HMD-based VR content well”). Each item in the instrument is scored on a 5-point Likert scale (1, “not at all” to 5, “very much so”), with higher mean scores indicating higher self-efficacy for using HMD-based VR content (possible range, 1–5 points). The reliability of the instrument was shown by a Cronbach’s α of .77 in the study by Park et al. [13] and .86 in the present study.

**Innovative propensity:** To measure the level of novelty-seeking and accepting change, three items from the “Innovative propensity regarding smartphone app scale” developed by Park et al. [13] were partially revised in accordance with the objective of the present study (i.e., revised to “I tend to use new devices or content earlier than others”). Each item in the instrument is scored on a 5-point Likert scale (1, “not at all” to 5, “very much so”), with higher mean scores indicating higher innovative propensity (possible range, 1–5 points). The reliability of the instrument was shown by a Cronbach’s α of .86 in the study by Park et al. [13] and .84 in the present study. In addition to subscale scores, a total score was calculated (possible range, 1–5 points) for this study.

#### Social factors

**Influential others:** To measure the influence of others on participants’ use of HMD-based VR content, three items from the four-item “Social influence on healthcare app by smartphone scale” developed by Sim et al. [14] were revised in accordance with the objective of the present study (i.e., revised to “People who have an influence on me believe I should use HMD-based VR content”). Each item in the instrument developed by Sim et al. [14] is scored on a 5-point Likert scale (1, “not at all” to 5, “very much so”), with higher mean scores indicating greater encouragement from people (possible range, 1–5 points). The reliability of the instrument was shown by a Cronbach’s α of .86 in the study by Sim et al. [14] and .91 in the present study.

**Environmental support:** To measure the degree to which the environment supports the use of HMD-based VR content, one item consisting of “My surrounding environment mostly supports my use of HMD-based VR content” was scored on a 5-point Likert scale (1, “not at all” to 5, “very much so”), with higher scores indicating higher level of environmental support.

In addition to subscale scores, a total score was calculated (possible range, 1-5 points) for this study.

#### Virtual reality content factors

**Engagement:** The level of engagement related to entertainment, interest, customization, interactivity, and target group of the content was assessed. Among 20 items in the Mobile App Rating Scale developed by Stoyanov et al. [15] for assessing engagement, functionality, aesthetics, information, and subjective quality of mobile apps, five items were revised in accordance with the objective of the present study (i.e., revised to “Is the HMD-based VR content entertaining to use?”). Each item in the instrument is scored on a 5-point Likert scale (1, “not at all” to 5, “very much so”), with higher mean scores indicating a more positive perception of content engagement (possible range, 1–5 points). The reliability of the engagement subscale of the instrument was shown by a Cronbach’s α of .89 in the study by Stoyanov et al. [15] and .89 in the present study.

**Functionality:** The level of functionality related to performance, ease of use, navigation, and gestural design of the content were assessed. Four items related to functionality in the study of Stoyanov et al. [15] were revised for the present study (i.e., revised to “How easy is it to learn how to use the HMD-based VR content?”). Each item in the instrument is scored on a 5-point Likert scale (1, “not at all” to 5, “very much so”), with higher mean scores indicating more positive perception of content functionality (possible range, 1–5 points). The reliability of the functionality subscale of the instrument was shown by a Cronbach’s α of .80 in the study by Stoyanov et al. [15] and.91 in the present study.

**Aesthetics:** The level of aesthetics related to the layout, graphics, and visual appeal of the content was assessed. Three items related to aesthetics in the study of Stoyanov et al. [15] were revised for the present study (i.e., revised to “Is arrangement and size of buttons/icons/menus/content on the screen appropriate?”). Each item in the instrument is scored on a 5-point Likert scale (1, “not at all” to 5, “very much so”), with higher mean scores indicating more positive perception of content aesthetics (possible range, 1–5 points). The reliability of the aesthetics subscale of the instrument was shown by a Cronbach’s α of .86 in the study by Stoyanov et al. [15] and.84 in the present study.

**Presence:** To measure presence (i.e., the feeling of being within an environment mediated by media), six items from the presence scale used by Lu [16] were partially revised in accordance with the objective of the present study (i.e., revised to “Once I finished using the VR content, I felt like I’d returned to reality after completing a trip”). Each item in the instrument is scored on a 5-point Likert scale (1= “not at all” to 5= “very much so”), with higher mean scores indicating higher presence (possible range, 1-5 points). The study by Lu [16] did not report the reliability of the scale. In the present study, the reliability of the six-item presence scale was shown by a Cronbach’s α of .80. In addition to subscale scores, a total score was calculated (possible range, 1-5 points) for this study.

#### Head-mounted display-related factors

**Cyber sickness:** To measure the symptom of motion sickness that occurs during VR experience, 16 items from the Simulator Sickness Questionnaire developed by Kennedy et al. [17] were used (i.e., “I felt general discomfort”). Each item in the instrument is scored on a 5-point Likert scale (1, “no symptoms” to 5, “severe symptoms”), with higher mean scores indicating higher cyber sickness (possible range, 1–5 points). The reliability of the scale was not reported by the developer [17] but was shown to be good by a Cronbach’s α of .95 was in the present study.

**Physical discomfort:** To measure the physical discomfort participants felt when wearing HMD, seven items were developed by the research team based on the results of the study by Eoh et al. [18], which measured discomfort when wearing a face mask and glasses (i.e., developed and used “I felt my nose being pressed when wearing an HMD”). The appropriateness of the questions was assessed through expert and face validity testing with five experts in HMD-VR research and technology development. Each item in the instrument is scored on a 5-point Likert scale (1, “no discomfort” to 5, “severe discomfort), with higher mean scores indicating higher physical discomfort (possible range, 1–5 points). The reliability of the physical discomfort scale used in the present study was shown by a Cronbach’s α of .88. In addition to subscale scores, a total score was calculated (possible range, 1–5 points) for this study.

#### Sociodemographic characteristics

The following characteristics were assessed (nine items): age, sex, average number of HMD-based VR content usage, total HMD-based VR usage period, experience using entertainment VR content, experience using education VR content, experience using healthcare VR content, intention to purchase HMD-based VR content, and intention to purchase HMD.

### Data analysis

The collected data were analyzed using IBM SPSS ver. 19.0 (IBM Corp., Armonk, NY, USA). Major variables were checked for a normal distribution (Kolmogorov-Smirnov test), and two-tailed *p*-values of <.05 were considered significant. The general characteristics of the participants and related variables were expressed as frequency, percentage, mean, and standard deviation. The reliability of the variables was quantified using Cronbach’s α. Differences in continuous usage intention of HMD-based VR according to the participants’ characteristics were analyzed using parametric tests (independent t-test, one-way analysis of variance) and nonparametric tests (Mann-Whitney U-test, Kruskal-Wallis test) in consideration of the normality of the data distribution. Correlations were computed using Pearson correlations. The influence of the characteristics of the participants and personal cognition, social, VR content, and HMD-related factors on continuous usage intention of HMD-based VR content was analyzed using hierarchical multiple regression analysis. Before performing hierarchical multiple regression analysis, the regression model was constructed after confirming the absence of multicollinearity between the variables.

## Results

### Participants’ characteristics and differences in continuous usage intention of head-mounted display-based virtual reality content

The majority of individuals with experience using HMD-based VR were 20 to 29 years of age (71.0%), with 57.6% being male. When asked about the frequency of VR usage, the most common response was 1 or 2 times, accounting for 64.5% of responses. The most common duration of total VR usage was less than 6 months (91.7%). Conversely, the least common frequency of VR usage was 6 times, reported by only 2.8% of respondents. Similarly, a total VR usage period of 12 months or more was the least common response (also 2.8%). An overwhelming majority of participants (98.6%) reported having used VR for entertainment purposes. However, only a small proportion of participants had experience using VR for educational (6.5%) and healthcare (3.2%) purposes. Meanwhile, 25.8% and 25.3% of participants expressed an intention to purchase HMD-based VR content and HMDs, respectively. The continuous usage intention of HMD-based VR content was significantly higher among those aged 10 to 19 years (*p*<.001), males (*p*<.001), those with experience using healthcare content (*p*=.019), those intending to purchase HMD-based VR content (*p*<.001), and those intending to purchase HMDs (*p*<.001) (Table 1).

### Level of continuous usage intention of head-mounted display-based virtual reality content and personal cognition, social, virtual reality content, and head-mounted display-related factors

The mean score for continuous usage intention of HMD-based VR content was close to the mid-point of the scale, at 2.59±0.57. The self-efficacy score was 2.49±0.59 points, the innovative propensity score was 2.51±0.64 points, and the total score for personal cognition factors was 2.50±0.57 points. The influential others score was 3.02±0.99 points, the environmental support score was 3.12±1.12 points, and the total score for social factors was 3.07±0.99 points. The engagement score was 3.61±0.77 points, the functionality score was 3.55±0.83 points, the aesthetics score was 3.55±0.78 points, the presence score was 3.26±0.83 points, and the total score for VR content factors was 3.49±0.67 points. The total score for HMD-related factors was 2.38±0.74 points, while that for cyber sickness was 2.17±0.80 points and the mean physical discomfort score was 2.59±0.90 points (Table 2).

### Distribution of personal cognition, social, virtual reality content, and head-mounted display-related factors according to the experience of using virtual reality content by type

**Figure 1.**
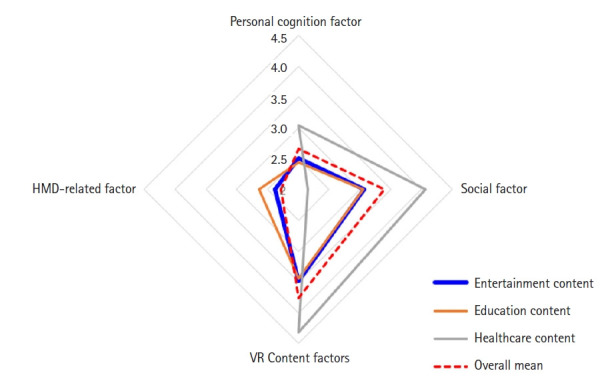
Distribution of personal cognition, social, VR content, and HMD-related factors according to the experience of using VR content by type. HMD, head-mounted display; VR, virtual reality.

The scores for personal cognition, social, and VR content factors were 2.51±0.56, 3.07±0.99, and 3.49±0.66 points among those with experience using VR content for entertainment, respectively, and 2.44±0.62, 3.06±1.11, and 3.44±0.80 points among those with experience using VR content for education. Conversely, participants who had experience using VR content in healthcare settings demonstrated notably higher scores of 3.04±0.55, 4.07±0.97, and 4.32±0.50 points, respectively. Regarding HMD-related factors, those with experience using healthcare VR content had the lowest score of 1.84±0.79 points. However, individuals who had used VR content for entertainment and education had higher scores, with 2.38±0.73 and 2.64±1.04 points, respectively (Figure 1).

### Correlations between continuous usage intention of head-mounted display-based virtual reality content and study variables

The participants’ continuous usage intention of HMD-based VR content showed statistically significant positive correlations with self-efficacy (r=0.59, *p*<.001), innovative propensity (r=0.46, *p*<.001), influential others (r=0.52, *p*<.001), environmental support (r=0.42, *p*<.001), engagement (r=0.67, *p*<.001), functionality (r=0.57, *p*<.001), aesthetics (r=0.47, *p*<.001), and presence (r=0.35, *p*<.001). However, the continuous usage intention of HMD-based VR content demonstrated statistically significant, albeit weak, negative correlations with cyber sickness (r=–0.29, *p*<.001) and physical discomfort (r=–0.14, *p*=.039) (Table 3).

### Factors influencing continuous usage intention of head-mounted display-based virtual reality content

Model 1, which incorporated personal cognition factors, explained approximately 35.1% of variance in the continuous usage intention of HMD-based VR content (F=57.962, *p*<.001). Self-efficacy (β=.532, *p*<.001) was identified as a variable with significant influence. When social factors were added in model 2, the explanatory power for the continuous usage intention of HMD-based VR content rose to approximately 39.5% (F=34.62, *p*<.001). This suggests that social factors contributed an additional explanatory power of approximately 4.4%. In model 2, both self-efficacy (β=.46, *p*<.001) and influential others (β=.32, *p*<.001) were identified as variables with significant influence. In model 3, the inclusion of VR content factors increased the explanatory power for the continuous usage intention of HMD-based VR content to approximately 50.2% (F=26.19, *p*<.001). This indicates that VR content factors contributed an additional explanatory power of approximately 10.7%. In this model, self-efficacy (β=.18, *p*=.039), influential others (β=.30, *p*<.001), and engagement (β=.50, *p*<.001) were identified as variables with significant influence. Finally, in model 4, the addition of HMD-related factors increased the explanatory power for the continuous usage intention of HMD-based VR content to approximately 52.7% (F=22.98, *p*<.001). This suggests that HMD-related factors contributed an additional explanatory power of approximately 2.5%. In model 4, influential others (β=.37, *p*<.001), environmental support (β=–.18, *p*=.030), engagement (β=.45, *p*<.001), and cyber sickness (β=–.21, *p*=.001) were identified as variables with significant influence (Table 4).

## Discussion

Investigating the continuous usage intention among users is crucial for achieving a product’s goals and securing a competitive market advantage [18]. However, the continuous usage intention of HMD-based VR content among users is not currently well understood. Therefore, this study aims to provide foundational data to support the expansion and growth of HMD-based VR content in the healthcare market.

This study found that the continuous usage intention for HMD-based VR content was at the mid-point (2.59 points). This may be attributed to the participants’ negative perception of their experiences. This conclusion is supported by the low usage rate, with 64.5% of respondents using it only 1 or 2 times, and the short usage duration, with 91.7% of respondents using it for less than 6 months. Furthermore, only about a quarter of the participants expressed an intention to purchase HMD-based VR content and HMDs, indicating a low inclination to make a personal investment in HMD-based VR, which reaffirms their negative perception of their experiences. The continuous usage intention of a specific product or service is determined by user satisfaction [19,20]. Therefore, to encourage the continuous usage of HMD-based VR content, efforts must be made from various angles to enhance user satisfaction. In the healthcare field, HMD-based VR content is being developed and utilized for staff training, patient education, and patient management. Continuous usage, as opposed to one-time usage, is crucial to achieve these objectives [3,4]. Therefore, not only content development but also a strategic approach to foster interest and enjoyment, which will encourage continuous usage, must be considered.

There were positive correlations between personal cognition factors (self-efficacy and innovative propensity) and the continuous usage intention of HMD-based VR content. This correlation also demonstrates a high explanatory power for continuous usage intention, accounting for 35.1% of the variance. Notably, models 1, 2, and 3 from the hierarchical multiple regression analysis indicate that self-efficacy is a significantly influential variable, warranting further attention. Self-efficacy in relation to a specific information technology refers to the confidence in one’s ability to readily adopt and utilize the technology without hesitation [21]. The low self-efficacy score (2.49) in this study suggests that participants found it challenging and had a negative perception of using HMD-based VR content. Therefore, to enhance the continuous usage intention of HMD-based VR content, it is necessary to implement specific strategies that can help users understand and learn how to use the technology more easily. These strategies could include user manuals and instructional videos. Additionally, future research should aim to identify the specific challenges users may encounter when using HMD-based VR content. This research should be conducted from the user’s perspective, rather than the technology developer’s perspective, to uncover potential solutions.

Social factors, along with the influence of others and environmental support, demonstrated a significant positive correlation with continuous usage intention. However, these factors only accounted for 4.4% of the explanatory power. In the hierarchical multiple regression analysis, models 2, 3, and 4 indicated that the influence of others is a significant variable, warranting careful attention. Social factors play a role in the adoption and sustained use of new information technology [22]. As such, strategies that reinforce encouragement and support from peers and content operators can enhance the intention for continuous use, rather than solely depending on user willingness. Specifically, for healthcare content that necessitates expert medical knowledge to promote health, strategies should be implemented to enable relevant experts to provide information, recommend usage, and encourage continued use.

The factors of engagement, functionality, aesthetics, and presence, all of which are elements of VR content, demonstrated significantly positive correlation with continuous usage intention. However, their explanatory power was limited to only 10.7%. Hierarchical multiple regression models 3 and 4 confirmed that engagement is a significant influencing variable. Conversely, presence, previously identified in studies as a key variable for the success likelihood of VR content in the market [23,24], did not exhibit statistical significance in this study. These findings suggest that while presence may offer enjoyment and an incentive to begin using VR content, it alone cannot induce sustained usage. Therefore, to foster continuous usage intention, the characteristics of the target group should be taken into account from the content development stage. This includes incorporating strategies that can continually enhance engagement, such as entertainment and interest.

As HMD-related factors, cyber sickness and physical discomfort showed significant negative weak correlations with the continuous usage intention of HMD-based VR content, with an explanatory power of only 2.5%. The physical adverse effects of HMD, such as cyber sickness, act as a major deterrent to the use of HMD-based VR [8]. The low incidence of cyber sickness reported in this study may be due to the fact that the questionnaire was not administered immediately following HMD use. Cyber sickness is a critical issue that needs to be addressed to encourage more active VR use [2,6]. It was also identified as a significant influencing variable in the hierarchical multiple regression analysis of model 4. Therefore, when considering continuous usage intention, it’s important to take into account the characteristics of the target group, such as their health status and age, from the content development stage. This allows for the adjustment of factors that could induce cyber sickness, including movement, graphics, and the visual appeal of content. Implementing restrictions on content usage time and device application methods may also be beneficial.

This study had the following limitations. First, the continuous usage intention of HMD-based VR content in the healthcare field was assessed by examining basic variables such as self-efficacy, innovative propensity, and social support, due to a lack of prior studies related to VR content in healthcare. As such, further investigation into associations with various health-related variables and influencing factors is necessary. Second, the participants ranged in age from 10 to 39 years, with more than half being male, and only 3.2% having experience using healthcare VR content. This limits the generalizability of the findings to groups who are not familiar with this technology. In future studies, the distribution of age and experience with healthcare VR content should be taken into account when selecting participants. Studies that include a broader age range and focus on healthcare VR content use will provide more specific insights into maintaining usage intention for health promotion.

Nevertheless, this study explored the continuous usage intention of healthcare content by examining personal cognition, social, VR content, and HMD-related factors. In conclusion, this study found that participants who had experience with healthcare content scored higher in areas of personal cognition, social aspects, and VR content compared to those without such experience. Interestingly, they scored lower in HMD-related factors. Therefore, to effectively employ HMD-based VR content in health education, it would be beneficial to simplify the content for ease of use and provide comprehensive instructions on how to use it. From a social standpoint, crafting expert guidance on content usage and promoting its continued use could prove beneficial. In terms of VR content, creating and supplying engaging and captivating strategies that take into account user characteristics could be advantageous. Regarding HMDs, it would be prudent to preemptively test for factors that could potentially lead to physical side effects such as cyber sickness. Developing content that considers performance, graphics, and usage time that could trigger such adverse effects is also recommended. Furthermore, it would be essential to devise strategies to address any issues that may arise.

## Figures and Tables

**Table 1.** Participants’ characteristics and differences in continuous usage intention of HMD-based VR content (N=217)

Characteristics	Categories	n (%)	Continuous use intention of HMD-based VR content, Mean±SD	F/H/t/U (*p*)
Age (year)	10s	56 (25.8)	2.92±0.53	23.43 (<.001)
	20s	154 (71.0)	2.48±0.58	
	30s	7 (3.2)	2.28±0.41	
Sex	Male	125 (57.6)	2.76±0.60	3.86 (<.001)
	Female	92 (42.4)	2.46±0.55	
Average number of times using HMD-based VR content	1–2	140 (64.5)	2.54±0.61	4.33 (.115)
	3–5	71 (32.7)	2.66±0.55	
	≥6	6 (2.8)	2.94±0.56	
Total HMD-based VR usage period (month)	>6	199 (91.7)	2.59±0.60	0.20 (.907)
	6–12	12 (5.5)	2.54±0.54	
	≥12	6 (2.8)	2.70±0.51	
Experience using VR content for entertainment	Yes	214 (98.6)	2.59±0.58	387.50 (.533)
	No	3 (1.4)	2.38±1.09	
Experience using VR content for education	Yes	14 (6.5)	2.53±0.60	1,299.50 (.589)
	No	203 (93.5)	2.59±0.59	
Experience using VR content for healthcare	Yes	7 (3.2)	3.10±0.48	1,113.00 (.019)
	No	210 (96.8)	2.57±0.59	
Intention to purchase HMD-based VR content	No	81 (37.3)	2.28±0.61	29.05 (<.001)
	Not sure	80 (36.9)	2.64±0.47	
	Yes	56 (25.8)	2.97±0.47	
Intention to purchase HMD	No	92 (42.4)	2.28±0.55	30.67 (<.001)
	Not sure	70 (32.3)	2.71±0.50	
	Yes	55 (25.3)	2.95±0.49	

HMD: head-mounted display, VR: virtual reality. F (*p*): Test statistic and *p*-value obtained from one-way analysis of variance. H (*p*): Test statistic and *p*-value obtained from the Kruskal-Wallis test. t (p): Test statistic and *p*-value obtained from independent t-test. U (*p*): Test statistic and *p*-value obtained from the Mann-Whitney U-test.

**Table 2.** Mean scores for continuous usage intention of HMD-based VR content and its influencing factors (N=217)

Variable	Mean±SD^†^
Continuous usage intention of HMD-based VR content	2.59±0.59
Personal cognition factors	2.50±0.57
Self-efficacy	2.49±0.59
Innovative propensity	2.51±0.64
Social factors	3.07±0.99
Influential others	3.02±0.99
Environmental support	3.12±1.12
VR content factors	3.49±0.67
Engagement	3.61±0.77
Functionality	3.55±0.83
Aesthetics	3.55±0.78
Presence	3.26±0.83
HMD-related factors	2.38±0.74
Cyber sickness	2.17±0.80
Physical discomfort	2.59±0.90

HMD, head-mounted display; VR, virtual reality.

† Possible range, 1–5.

**Table 3.** Pearson correlation coefficients between factors and continuous usage intention of HMD-based VR content (N=217)

Factor	Categories	r (*p*)
Personal cognition factors	Self-efficacy	.59 (<.001)
	Innovative propensity	.46 (<.001)
Social factors	Influential others	.52 (<.001)
	Environmental support	.42 (<.001)
VR content factors	Engagement	.67 (<.001)
	Functionality	.54 (<001)
	Aesthetics	.47 (<.001)
	Presence	.35 (<.001)
HMD-related factors	Cyber sickness	–.29 (<.001)
	Physical discomfort	–.14 (.039)

HMD, head-mounted display; VR, virtual reality r (*p*): Test statistic and *p*-value obtained from Pearson correlation test.

**Table 4.** Factors Influencing Continuous Usage Intention of HMD-based VR Content (N=217)

Model	Predictors	Categories	β	t (*p*)	R^2^	Adj R^2^	ΔAdj R²	F (*p*)
1	(Constant)			7.27 (<.001)	.59	.35	.35	57.96 (<.001)
	Personal cognition factors	Self-efficacy	.53	6.91 (<.001)				
		Innovative propensity	.08	1.08 (.28)				
2	(Constant)			7.27 (<.001)	.63	.40	.04	34.62 (<.001)
	Personal cognition factors	Self-efficacy	.46	5.64 (<.001)				
		Innovative propensity	–.013	–0.17 (.87)				
	Social factors	Influential others	.32	3.54 (<.001)				
		Environmental support	–.08	–0.98 (.33)				
3	(Constant)			3.88 (<.001)	.71	.50	.11	26.19 (<.001)
	Personal cognition factors	Self-efficacy	.18	2.08 (.04)				
		Innovative propensity	–.02	–0.28 (.78)				
	Social factors	Influential others	.30	3.36 (<.001)				
		Environmental support	–.18	–2.17 (.03)				
	VR Content factors	Engagement	.50	5.71 (<.001)				
		Functionality	–.03	–0.30 (.76)				
		Aesthetics	.03	0.39 (.70)				
		Presence	–.03	–0.56 (.57)				
4	(Constant)			4.23 (<.001)	.77	.53	.03	22.98 (<.001)
	Personal cognition factors	Self-efficacy	.16	1.81 (.07)				
		Innovative propensity	–.05	–0.65 (.51)				
	Social factors	Influential others	.37	4.11 (<.001)				
		Environmental support	–.18	–2.19 (.03)				
	VR Content factors	Engagement	.45	5.18 (<.001)				
		Functionality	–.01	–0.10 (.92)				
		Aesthetics	.004	0.056 (.96)				
		Presence	–.03	–0.44 (.66)				
	HMD-related factors	Cyber sickness	–.21	–3.33 (.001)				
		Physical discomfort	.11	1.81 (.07)				

Adj., adjusted; HMD, head-mounted display; VR, virtual reality. F (*p*): Overall test statistic and *p*-value for the regression model.
